# Graphical approaches for multiple comparison procedures using weighted Bonferroni, Simes, or parametric tests

**DOI:** 10.1002/bimj.201000239

**Published:** 2011-08-12

**Authors:** Frank Bretz, Martin Posch, Ekkehard Glimm, Florian Klinglmueller, Willi Maurer, Kornelius Rohmeyer

**Affiliations:** 1 Statistical Methodology, Novartis Pharma AGBasel, Switzerland; 2 Section of Medical Statistics, Center for Medical Statistics, Informatics and Intelligent Systems, Medical University of ViennaVienna, Austria; 3 Institute of Biostatistics, University of HannoverHannover, Germany

**Keywords:** Dunnett test, Gatekeeping procedure, Min-*p* test, Non-inferiority, Truncated Holm

## Abstract

The confirmatory analysis of pre-specified multiple hypotheses has become common in pivotal clinical trials. In the recent past multiple test procedures have been developed that reflect the relative importance of different study objectives, such as fixed sequence, fallback, and gatekeeping procedures. In addition, graphical approaches have been proposed that facilitate the visualization and communication of Bonferroni-based closed test procedures for common multiple test problems, such as comparing several treatments with a control, assessing the benefit of a new drug for more than one endpoint, combined non-inferiority and superiority testing, or testing a treatment at different dose levels in an overall and a subpopulation. In this paper, we focus on extended graphical approaches by dissociating the underlying weighting strategy from the employed test procedure. This allows one to first derive suitable weighting strategies that reflect the given study objectives and subsequently apply appropriate test procedures, such as weighted Bonferroni tests, weighted parametric tests accounting for the correlation between the test statistics, or weighted Simes tests. We illustrate the extended graphical approaches with several examples. In addition, we describe briefly the gMCP package in R, which implements some of the methods described in this paper.

## 1 Introduction

Multiple test procedures are often used in the analysis of clinical trials addressing multiple objectives, such as comparing several treatments with a control and assessing the benefit of a new drug for more than one endpoint. Several multiple test procedures have been developed in the recent past that allow one to map the relative importance of the different study objectives as well as their relation onto an appropriately tailored multiple test procedure.

A common strategy to reduce the degree of multiplicity is to group the hypotheses into primary and secondary objectives (O'Neill, 1997). Test procedures accounting for the inherent logical relationships include fixed sequence tests (Maurer et al., 1995; Westfall and Krishen, 2001), gatekeeping procedures (Bauer et al., 1998; Westfall and Krishen, 2001; Dmitrienko et al., 2003) and fallback procedures (Wiens, 2003; Huque and Alosh, 2008). Li and Mehrotra (2008) introduced a more general approach for adapting the significance level to test secondary hypotheses based on the finding for the primary hypotheses. Alosh and Huque (2009) introduced the notion of consistency when testing for an effect in the overall population and in a specific subgroup. The authors extended this consistency concept to other situations (Alosh and Huque, 2010), including how to address multiplicity issues of a composite endpoint and its components in clinical trials (Huque et al., 2011). Hung and Wang (2009, 2010) considered some controversial multiple test problems, with emphasis on regulatory applications, and pointed out illogical problems that may arise with recently developed multiple test procedures.

In this paper, we focus on graphical approaches which have been introduced independently by Bretz et al. (2009) and Burman et al. (2009). The key idea is to express the resulting multiple test procedures by directed, weighted graphs, where each node corresponds to an elementary hypothesis, together with a simple algorithm to generate such graphs while sequentially testing the individual hypotheses. Using graphical approaches, one can explore different test strategies together with the clinical team and thus tailor the multiple test procedure to the given study objectives. So far, the description of these graphical approaches has focused on Bonferroni-based test procedures. In this paper, we investigate extensions of the original ideas. In particular, we discuss in Section 2 how a separation between the weighting strategy and the test procedure facilitates the application of a graphical approach beyond Bonferroni-based test procedures. In Section 3, we illustrate these ideas with different test procedures. We start with a brief review of Bonferroni-based test procedures and subsequently describe parametric graphical approaches that account for the correlation between the test statistics as well as graphical approaches using the Simes test. In Section 4, we describe the gMCP package in R which implements some of the methods discussed in this paper and illustrate it with a clinical trial example using a truncated Holm procedure. Concluding remarks are given in Section 5.

## 2 Graphical weighting strategies

Consider the problem of testing *m* elementary hypotheses *H*_1_,…,*H*_*m*_, some of which could be more important than others, e.g. primary and secondary objectives. Let 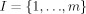 denote the associated index set. The closure principle introduced by Marcus et al. (1976) is commonly used to construct powerful multiple test procedures. Accordingly, we consider all non-empty intersection hypotheses 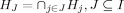. We further pre-specify an α-level test for each *H*_*J*_. The resulting closed test procedure rejects 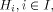 if all intersection hypotheses *H*_*J*_ with 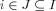 are rejected by their corresponding α-level tests. By construction, closed test procedures control the familywise error rate (FWER) in the strong sense at level α∈(0,1). That is, the probability to reject at least one true null hypothesis is bounded by α under any configuration of true and false null hypotheses (Hochberg and Tamhane, 1987). In fact, closed test procedures have certain optimality properties whenever the FWER has to be controlled (Bauer, 1991). In what follows, we assume that the hypotheses *H*_1_,…,*H*_*m*_ satisfy the free combination condition (Holm, 1979). If this condition is not satisfied, the methods in this paper still control the FWER at level α, although they can possibly be improved because of the reduced closure tree (Brannath and Bretz, 2010).

One important class of closed test procedures is obtained by applying weighted Bonferroni tests to each intersection hypothesis *H*_*J*_. For each *J*⊆*I* assume a collection of weights *w*_*j*_ (*J*) such that 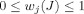 and 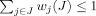. With the weighted Bonferroni test we reject *H*_*J*_ if 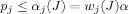 for at least one *j*∈*J*, where *p*_*j*_ denotes the unadjusted *p*-value for *H*_*j*_. Hommel et al. (2007) introduced a useful subclass of sequentially rejective Bonferroni-based closed test procedures. They showed that the monotonicity condition


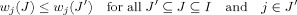
(1)

ensures consonance, i.e. if an intersection hypothesis *H*_*J*_ is rejected, there is an index *j*∈*J*, such that the elementary hypothesis *H*_*j*_ can be rejected as well. This substantially simplifies the implementation and interpretation of related closed test procedures, as the closure tree of 2*^m^*−1 intersection hypotheses is tested in only *m* steps. Many common multiple test procedures satisfy (1), see Hommel et al. (2007) for examples.

**Figure 1:**
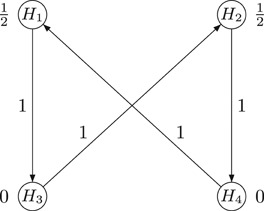
Weighting strategy for two hierarchically ordered endpoints and two dose levels.

Bretz et al. (2009) and Burman et al. (2009) independently derived graphical representations and associated rejection algorithms for important subclasses of the Hommel et al. (2007) procedures. The graphical representations and rejection algorithms in these two articles are different, though underlying ideas are closely related; see Guilbaud and Karlsson (2011) for some comparative examples. Using the graphical approach of Bretz et al. (2009), the hypotheses *H*_1_,…,*H*_*m*_ are represented by vertices with associated weights denoting the local significance levels α_1_,…,α_*m*_. In addition, any two vertices *H*_*i*_ and *H*_*j*_ are connected through directed edges, where the associated weight *g*_*ij*_ indicates the fraction of the (local) significance level α_*i*_ that is propagated to *H*_*j*_ once *H*_*i*_ (the hypothesis at the tail of the edge) has been rejected. A weight *g*_*ij*_=0 indicates that no propagation of the significance level is foreseen and the edge is dropped for convenience. Figure 1 shows an example.

While the original graphical approaches were introduced based on weighted Bonferroni tests, we propose here to dissociate the underlying *weighting strategy* from the employed *test procedure*. The benefit of such an approach is the enhanced transparency by (i) first deriving suitable weighting strategies that reflect the given study objectives (and which can be communicated to the clinical team) and (ii) subsequently applying appropriate test procedures that do not necessarily have to be based on Bonferroni's inequality.

Graphical weighting strategies are conceptually similar to the graphs proposed by Bretz et al. (2009). They essentially summarize the complete set of 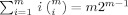 weights determining the full closure tree. A weighted multiple test can then be applied to each intersection hypothesis *H*_*J*_, such as a weighted Bonferroni test, a weighted min-*p* test accounting for the correlation between the test statistics, or a weighted Simes test; see Section 3 for details. Weighting strategies are formally defined through the weights *w*_*i*_ (*I*), *i*∈*I*, for the global null hypothesis *H*_*I*_ and the transition matrix **G**=(*g*_*ij*_), where 0≤*g*_*ij*_≤1, *g*_*ii*_=0, and 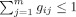 for all *i*, *j*∈*I*. We additionally need to determine how the graph is updated once a vertex is removed. This can be achieved by tailoring Algorithm 1 in Bretz et al. (2009) to the graphical weighting strategies as follows. For a given index set *J*⊆*I*, let *J^c^*=*I*\*J* denote the set of indices that are not contained in *J*. Then the following algorithm determines the weights *w*_*j*_(*J*), *j*∈*J*. This algorithm has to be repeated for each *J*⊆*I* to generate the 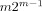 weights for the full closure.

**Algorithm 1 (Weighting Strategy)**

- Select *j*∈*J^c^* and remove *H*_*j*_
- Update the graph:
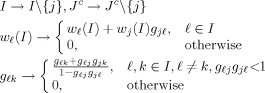
- If ∣*J^c^*∣≥1, go to step (i); otherwise 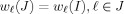, and stop.

As shown by Bretz et al. (2009), the weights *w*_*j*_(*J*), *j*∈*J* are unique. In particular, they do not depend on the sequence in which hypotheses 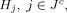 are removed in step (i) of Algorithm 1. Note that Algorithm 1 requires specifying the weights *w*_*j*_(*I*) for the global intersection hypothesis *H*_*I*_ and the elements of the transition matrix **G**. This leads to the specification of 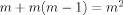 parameters if 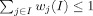 and 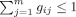 or 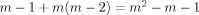 parameters if 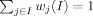 and 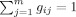, for all *i*, *j*∈*I*.

**Example 1**

As an example, assume a primary family of two hypotheses 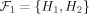 and a secondary family of two hypotheses 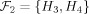. The hypotheses *H*_1_ and *H*_2_ could denote, for example, the comparison of low and high dose with a control, for either a primary endpoint, a non-inferiority claim, or an overall population. Accordingly, the hypotheses *H*_3_ and *H*_4_ would then denote the comparison of the same two doses with a control, for either a secondary endpoint, a superiority claim, or a pre-specified subgroup. Figure 1 visualizes one possible weighting strategy. It is motivated by a strict hierarchy within dose: the secondary endpoint will only be assessed if efficacy was shown previously for the primary endpoint (so-called successiveness property; see Maurer et al., 2011). If for one of the doses efficacy can be shown for both the primary and the secondary endpoint, the associated weight is passed on to the other dose. Therefore we have 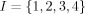, 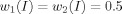 for the primary hypotheses and 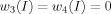 for the secondary hypotheses, which implies that no secondary hypothesis can be rejected until a primary hypothesis is rejected and propagates its weight. The associated transition matrix is


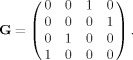


**Figure 2:**
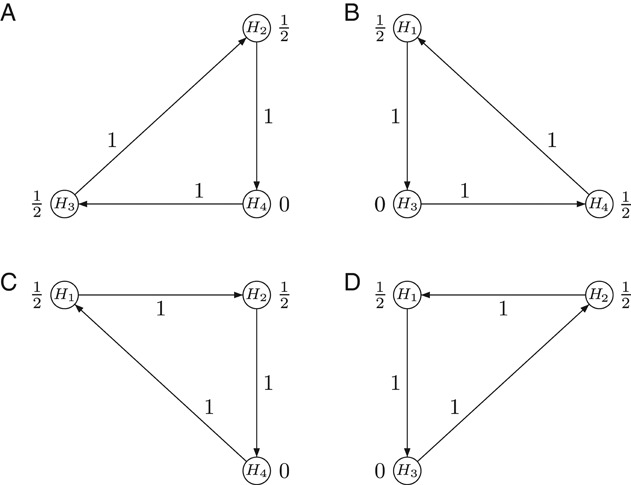
Updated graphs resulting from Figure 1 after removing (A) *H*_1_, (B) *H*_2_, (C) *H*_3_, and (D) *H*_4_.

The graph in Figure 1 together with Algorithm 1 from above fully specify the 32 weights of the full closure tree, as summarized in Table 1. This table parallels the weight tables introduced by Dmitrienko et al. (2003). Note that the weights 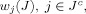 are formally not defined and expressed by “–” in Table 1. Figure 2 displays the updated graphs resulting from Figure 1 after removing *H*_1_, *H*_2_, *H*_3_, or *H*_4_. The four updated graphs in Figure 2 correspond to the four rows in Table 1 containing the weights for the three-way intersection hypotheses. Removing any two hypotheses results in six possible two-way intersection hypotheses and the two vertexes are connected by two directed edges, each with weight 1 (graphical display omitted here). Note that Figure 2 displays the principle of recalculating the weights by updating the graphs. It is possible and also necessary to remove hypotheses with weight 0 (in this example *H*_3_ and *H*_4_ with 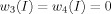) in order to compute the respective weights for the larger intersection hypotheses.

**Table 1:** Weights for the intersection hypotheses derived from Figure 1

Intersection hypothesis	Weights			
	
	*H*_1_	*H*_2_	*H*_3_	*H*_4_
*H*_1_∩*H*_2_∩*H*_3_∩*H*_4_	0.5	0.5	0	0
*H*_1_∩*H*_2_∩*H*_3_	0.5	0.5	0	–
*H*_1_∩*H*_2_∩*H*_4_	0.5	0.5	–	0
*H*_1_∩*H*_2_	0.5	0.5	–	–
*H*_1_∩*H*_3_∩*H*_4_	0.5	–	0	0.5
*H*_1_∩*H*_3_	1	–	0	–
*H*_1_∩*H*_4_	0.5	–	–	0.5
*H*_1_	1	–	–	–
*H*_2_∩*H*_3_∩*H*_4_	–	0.5	0.5	0
*H*_2_∩*H*_3_	–	0.5	0.5	–
*H*_2_∩*H*_4_	–	1	–	0
*H*_2_	–	1	–	–
*H*_3_∩*H*_4_	–	–	0.5	0.5
*H*_3_	–	–	1	–
*H*_4_	–	–	–	1

Note that Figure 1 displays only one possible weighting strategy. Many other weighting strategies are possible and perhaps more reasonable, depending on the given context. We refer to Bretz et al. (2011) for a generic discussion about testing two families 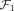 and 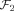 with two hypotheses each.

## 3 Test procedures

In Section 2, we proposed to dissociate the underlying weighting strategy from the employed test procedure and gave a generic description of the former, illustrated with an example. In this section we give details on different test procedures that could be employed to test the intersection hypotheses, including weighted Bonferroni tests, weighted min-*p* tests accounting for the correlation between the test statistics, and weighted Simes' tests.

### 3.1 Weighted Bonferroni tests

The weighted Bonferroni test introduced in Section 2 is the simplest applicable test procedure, leading to the original graphical approaches by Bretz et al. (2009). Applying the Bonferroni test leads to simple and transparent test procedures that are often easier to communicate than alternative, potentially more powerful approaches. As a matter of fact, the Bonferroni test is often perceived to provide credible trial outcomes in clinical practice. Most importantly in the context of the graphical weighting strategies considered here, applying the Bonferroni test leads to shortcut procedures as long as the monotonicity condition (1) is satisfied. That is, one can start with a graph as shown in Figure 1 and sequentially test the *m* hypotheses as long as individual null hypotheses *H*_*i*_, *i* ∈*I*, are rejected. Based on Algorithm 1 from Section 2, we give in the following a similar algorithm that accounts for the weighted Bonferroni tests, thus leading to the sequentially rejective multiple test procedures described in Bretz et al. (2009):

**Algorithm 2 (Weighted Bonferroni Test)**

- Select a *j*∈*I* such that *p*_*j*_≤*w*_*j*_(*I*)α and reject *H*_*j*_; otherwise stop.
- Update the graph:
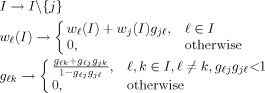
- If ∣*I*∣≥1, go to step (i); otherwise stop.

Similar to Algorithm 1, the results in Bretz et al. (2009) ensure that the decisions of the resulting sequentially rejective multiple test procedures remain unchanged regardless of the actual rejection sequence. That is, if in step (i) of Algorithm 2 more than one hypothesis could be rejected, it does not matter with which to proceed. Although Algorithms 1 and 2 have a similar update rule in step (ii), they differ in the way that the index sets are updated. While Algorithm 2 starts with the global index set *I* and reduces it sequentially as long as hypotheses are rejected, Algorithm 1 removes, for each *J*⊆*I*, consecutively all indices from *I* that are not contained in *J* until the set *J* is obtained. Note that performing a closed weighted Bonferroni test procedure using the weights from Algorithm 1 leads to exactly the same test decisions as performing a sequentially rejective multiple test procedure with Algorithm 2 based on the same starting weights.

**Figure 3:**
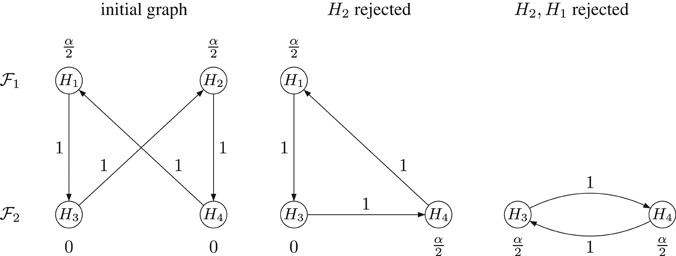
Graph for sequentially rejective procedure with example rejection sequence.

Figure 3 gives an example of a Bonferroni-based sequentially rejective multiple test procedures for the weighting strategy proposed in Example 1. Assume, for example, the unadjusted *p*-values *p*_1_=0.01, *p*_2_=0.005, *p*_3_=0.1, and *p*_4_=0.5. Then we can reject both *H*_1_ and *H*_2_, but none of the other hypotheses. Figure 3 displays the initial graph together with a possible rejection sequence. As mentioned above, the final decisions on which hypotheses to reject do not depend on the particular rejection sequence. That is, with the initial graph from Figure 3 we would obtain the same decisions, regardless of whether we first reject *H*_2_ and then *H*_1_, or vice versa.

Many standard approaches from the literature can be visualized using Bonferroni-based graphical test procedures, including the weighted or unweighted Bonferroni–Holm procedure (Holm, 1979), fixed sequence tests (Maurer et. al, 1995; Westfall and Krishen, 2001), fallback procedures (Wiens, 2003), and gatekeeping procedures (Bauer et al., 1998; Westfall and Krishen, 2001; Dmitrienko et al., 2003). Adjusted *p*-values and simultaneous confidence intervals can be calculated as well, although the resulting simultaneous confidence intervals are known to be of limited practical use, as they are often non-informative; see Strassburger and Bretz (2008), Guilbaud (2008, 2009) and Bretz et al. (2009) for details. Bretz et al. (2011) provided SAS/IML code to perform the resulting Bonferroni-based sequentially rejective multiple test procedures. In Section 4, we describe the gMCP package in R, which offers a convenient graphical user interface (GUI) for these approaches.

One general disadvantage of Bonferroni-based approaches is a perceived power loss, motivating the use of weighted parametric tests that account for the correlation between the test statistics or the use of weighted Simes tests. We discuss these alternative test procedures in Sections 3.2 and 3.3, respectively.

### 3.2 Weighted parametric tests

If for the intersection hypotheses 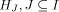, the joint distribution of the *p*-values *p*_*j*_, *j*∈*J*, are known, a weighted min-*p* test can be defined (Westfall and Young, 1993; Westfall et al., 1998). This test rejects *H*_*J*_ if there exists a *j*∈*J* such that 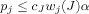, where *c*_*J*_ is the largest constant satisfying


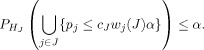
(2)

If the *p*-values are continuously distributed, there is a *c*_*J*_ such that the rejection probability is exactly α. Determination of *c*_*J*_ requires knowledge of the joint null distribution of the *p*-values and computation of the corresponding multivariate cumulative distribution functions. If the test statistics are multivariate normal or *t* distributed under the null hypotheses, these probabilities can be calculated using, for example, the mvtnorm package in R (Genz and Bretz, 2009). Alternatively, resampling-based methods may be used to approximate the joint null distribution; see Westfall and Young (1993).

If *c*_*J*_=1 in (2), the weighted parametric test reduces to the weighted Bonferroni test. This fully exhausts the level if and only if the joint distribution of continuously distributed *p*-values with strictly positive density function over (0,1)*^m^* satisfies


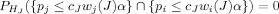


for all *i*≠*j*∈*J*, because then all events are pairwise disjoint and 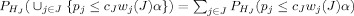. Otherwise, *c*_*J*_>1 and the weighted parametric test gives a uniform improvement over the weighted Bonferroni test from Section 3.1.

If not all, but some of the multivariate distributions of the *p*-values are known, it is possible to derive conservative upper bounds of the rejection probability that still give an improvement over the Bonferroni test. Assume that *I* can be partitioned into *l* sets *I*_*h*_ such that 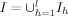 and 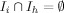 for 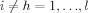. We assume that for each *h*=1,…,*l* the joint distribution of the *p*-values 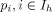, is known, but the joint distribution of *p*-values belonging to different *I*_*h*_ is not necessarily known. Now, let *J*⊆*I* and choose the maximal critical value *c*_*J*_ such that


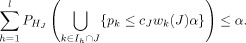
(3)

By the Bonferroni inequality, the left-hand side in (2), which cannot be computed if the full joint distribution is unknown, is bounded from above by the left-hand side in (3), whose computation requires only the knowledge of the joint distribution of the *p*-values in *I*_*h*_∩*J*, separately for each *h*=1,…,*l*. Thus, any *c*_*J*_ satisfying (3) will also satisfy (2), leading to a conservative test for the intersection hypothesis *H*_*J*_.

It follows immediately from Eq. (1) that these parametric approaches are consonant if


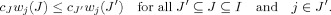
(4)

For *p*-values following a joint continuous distribution with strictly positive density function over (0,1)*^m^* this is also a necessary consonance condition. This condition is often violated by the weighted parametric tests above. Consider, for example, the Sidak (1967) test for three hypotheses with initial weights 1/3. Assume that for the test of the intersection of any two hypotheses the weights are 1/3 and 2/3. For α=0.05, the critical value *c*_*J*_*w*_*j*_ (*J*)α=0.01695 for all three hypotheses in the first step. For all *J*′ with ∣*J*′∣=2, we have 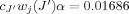 for the hypothesis *H*_*j*_ with the weight 1/3 in the second step, violating (4). This phenomenon is even more pronounced for positive correlations. If in the previous example the correlations are all 0.5 (corresponding to a Dunnett test in a balanced one-way layout with known variance), we have 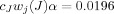 and 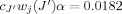.

If the consonance condition (4) is met, a sequentially rejective test procedure similar to the Bonferroni-based graphical tests from Section 3.1 can be defined.

**Algorithm 3 (Weighted Parametric Test)**

- Choose the maximal constant *c*_*I*_ that satisfies either (2) or (3) for *J*=*I*.
- Select a *j*∈*I* such that 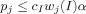 and reject *H*_*j*_; otherwise stop.
- Update the graph:
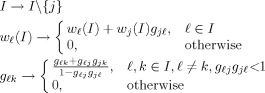
- If ∣*I*∣≥1, go to step (i); otherwise stop.

For any specific multiple test procedure defined by a given graph, the consonance condition can be checked. If the consonance condition is not met, the weighting strategies introduced in Section 2 remain applicable, although the connection to a corresponding sequentially rejective test procedure is lost. In this case, Algorithm 3 no longer applies and one has to go through the entire closed test procedure. For a given weighting strategy, this procedure is uniformly more powerful than the associated Bonferroni-based procedure from Section 3.1. Note that adjusted *p*-values for each hypothesis *H*_*i*_ can be obtained by computing *p*-values for each intersection hypothesis *H*_*J*_ with *i*∈*J* (given by the lowest local level for which the respective intersection hypothesis can be rejected) and then taking the maximum over them.

Before illustrating Algorithm 3 with two examples, we notice that Eq. (2) does not provide the only possible definition of a weighted parametric test. Instead of using *c*_*J*_*w*_*j*_(*J*)α as the critical values for *p*_*j*_, *j*∈*J*, we could also use some other function *f*_*J*_ (*w*_*j*_ (*J*),α) fulfilling 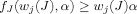 for all *j*∈*J* and all dependence structures of the *p*-values. For example, if 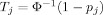 is the test statistic corresponding to the *p*-value of a *z*-test for *H*_*j*_, then finding an ε_*J*_ such that 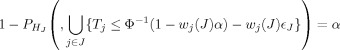 would also define a test which is uniformly more powerful than the corresponding weighted Bonferroni test. A related approach to account for correlations in weighted multiple testing procedures defined by the graphical approach was considered in Millen and Dmitrienko (2011).

**Example 2**

We revisit the weighting strategy from Example 1. Assume that the joint null distribution of the *p*-values *p*_1_, *p*_2_ for the two primary dose-control comparisons as well as the joint null distribution of the *p*-values *p*_3_, *p*_4_ for the two secondary comparisons are known. Applying the standard analysis-of-variance assumptions with a known common variance, we have a bivariate normal distribution, where the correlation is determined only by the relative group sample sizes. In practice, the correlation between primary and secondary endpoints is typically unknown and thus the joint distributions of the pairs 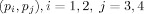 are also unknown. Therefore, (2) cannot be computed and *c*_*J*_ cannot be determined directly. Setting 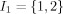 and 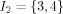, the joint null distribution of the test statistics for the hypotheses in *I*_1_ and *I*_2_ is known and the constants *c*_*J*_ can be determined by (3). Note that *c*_*J*_ depends on α and on the weights. Table 2 shows the local significance levels for both (A) the closed weighted Bonferroni test procedure and (B) the closed weighted parametric test procedure, assuming α=0.025 and equal group sample sizes.

**Table 2:** Local significance levels (in %) of A: weighted Bonferroni (B: parametric, C: consonant parametric with δ=0.0783) test for the example from Figure 1 and α=0.025

Intersection hypothesis	Local significance levels (in %)			
	
	*H*_1_	*H*_2_	*H*_3_	*H*_4_
*H*_1_∩*H*_2_∩*H*_3_∩*H*_4_	1.25 (1.35,1.35)	1.25 (1.35,1.35)	0 (0,0)	0 (0,0)
*H*_1_∩*H*_2_∩*H*_3_	1.25 (1.35,1.35)	1.25 (1.35,1.35)	0 (0,0)	–
*H*_1_∩*H*_2_∩*H*_4_	1.25 (1.35,1.35)	1.25 (1.35,1.35)	–	0 (0,0)
*H*_1_∩*H*_2_	1.25 (1.35,1.35)	1.25 (1.35,1.35)	–	–
*H*_1_∩*H*_3_∩*H*_4_	1.25 (1.25,1.35)	–	0 (0,0)	1.25 (1.25,1.15)
*H*_1_∩*H*_3_	2.50 (2.50,2.50)	–	0 (0,0)	–
*H*_1_∩*H*_4_	1.25 (1.25,1.35)	–	–	1.25 (1.25,1.15)
*H*_1_	2.50 (2.50,2.50)	–	–	–
*H*_2_∩*H*_3_∩*H*_4_	–	1.25 (1.25,1.35)	1.25 (1.25,1.15)	0 (0,0)
*H*_2_∩*H*_3_	–	1.25 (1.25,1.35)	1.25 (1.25,1.15)	–
*H*_2_∩*H*_4_	–	2.50 (2.50,2.50)	–	0 (0,0)
*H*_2_	–	2.50 (2.50,2.50)	–	–
*H*_3_∩*H*_4_	–	–	1.25 (1.35,1.35)	1.25 (1.35,1.35)
*H*_3_	–	–	2.50 (2.50,2.50)	–
*H*_4_	–	–	–	2.50 (2.50,2.50)

Using, for example, the mvtnorm package in R, one can call

> myfct <- function(x, a, w, sig) {

+ 1 - a - pmvnorm(lower = -Inf, upper = qnorm(1-x*w*a), sigma = sig)

+ }

> sig <- diag(2)*0.5 + 0.5

> uniroot(myfct, lower = 1, upper = 9, a = 0.025, w = rep(0.5, 2),

+ sig = sig)$root

[1] 1.078306

to compute *c*_*J*_=1.0783 for 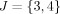 as well as for all 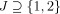 and *c*_*J*_=1 otherwise. In other words, *H*_3_∩*H*_4_ and all intersection hypotheses that include *H*_1_ and *H*_2_ are tested with unweighted Dunnett *z* tests. However, intersection hypotheses containing *H*_1_∩*H*_4_ or *H*_2_∩*H*_3_ are tested with an unweighted Bonferroni test. As a consequence, the resulting family of tests is not consonant. For example, 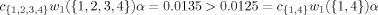, violating condition (4). Nevertheless, for a given weighting strategy, the closed test procedure based on parametric weighted tests dominates the associated procedure based on weighted Bonferroni tests. For example, if 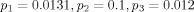, and *p*_4_=0.01, the weighted parametric test procedure rejects *H*_1_ and *H*_3_, whereas the Bonferroni test rejects none. In Section 4, we revisit this numerical example and describe the gMCP package in R, which implements the closed weighted parametric test procedure (B). Related gatekeeping procedures addressing the problem of comparing several doses with a control for multiple hierarchical endpoints were described, among others, by Dmitrienko et al. (2006), Liu and Hsu (2009), and Xu et al. (2009).

**Figure 4:**
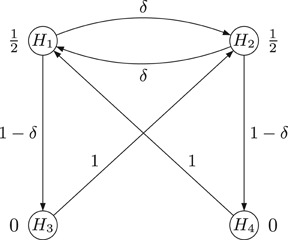
Graphical display of weighting strategy for a consonant weighted parametric test procedure.

Continuing with the example, one can enforce consonance via an appropriate modification of the weighting strategy from Figure 1. To achieve consonance, we introduce additional edges with weight δ (see Figure 4) such that the weight for *H*_1_ (resp. *H*_2_) is sufficiently increased to satisfy the monotonicity condition (4) when testing the intersection hypotheses *H*_1_∩*H*_4_ and *H*_1_∩*H*_3_∩*H*_4_ (resp. *H*_2_∩*H*_3_ and *H*_2_∩*H*_3_∩*H*_4_). If 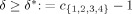 the resulting closed test procedure is consonant and Algorithm 3 can be used to perform the test. In the above example with α=0.025, the lower bound is δ^*^=0.0783. Setting δ=δ^*^, we obtain the local significance levels for procedure (C) in Table 2. Note that because of the special weighting strategy employed in this example, these local significance levels are obtained with the regular Dunnett and univariate *z* tests.

The lower bound δ^*^ depends on the correlation between the test statistics for *H*_1_ and *H*_2_. Because 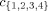 increases with the correlation, this also holds for δ^*^. In the limiting case that the sample size ratios of the dose groups and the control group tend to infinity, the correlation tends to 1. Consequently, 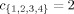, such that δ^*^=1 and the graph is degenerated for all α>0. On the other hand, if the above sample size ratios tend to 0, the correlation tends to 0 and 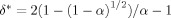 in limit.

Note that by enforcing consonance, the resulting multiple test procedure based on weighted parametric tests is no longer uniformly better than the associated Bonferroni-based test procedure which does not account for the correlations. That is, for a given weighting strategy, the closed test procedure based on parametric weighted tests may fail to reject certain hypotheses that otherwise are rejected by the associated procedure based on weighted Bonferroni tests. For example, if 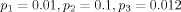, and *p*_4_=0.01, the initial graph from Figure 3 rejects *H*_1_ and *H*_3_, whereas the consonant weighted parametric test procedure from Figure 4 with δ=0.0783 rejects only *H*_1_.

**Example 3**

Consider again Example 1, but assume that *H*_1_,*H*_2_ are two non-inferiority hypotheses (say, for low and high dose against control) and *H*_3_,*H*_4_ are two superiority hypotheses (for the same two doses). We again make the standard analysis-of-variance assumptions with a known common variance and let α=0.025. Bonferroni-based graphical approaches for combined non-inferiority and superiority testing were described in Hung and Wang (2010) and Lawrence (2011). In the following, we exploit the fact that the correlations between the four test statistics are known. Therefore, the complete joint distribution is known and we can apply (2). Note that if *w*_*j*_ (*J*)=0 for some *j*∈*J*, the joint distribution degenerates. In our example it thus suffices to calculate bivariate or univariate normal probabilities.

Assume first that the same population is used for all four tests. For simplicity, assume further that the group sample sizes are equal. Then the correlation between the non-inferiority and superiority tests within a same dose is 1; all other correlations are 0.5. Therefore, *c*_*J*_=1.0783 for 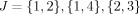, and 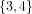. Otherwise, *c*_*J*_=1 and condition (4) is trivially satisfied. That is, consonance is ensured and one can apply Algorithm 3. This leads to a sequentially rejective multiple test procedure, where at each step either bivariate Dunnett *z* tests or individual *z* tests are used. This conclusion remains true if the common variance is unknown and Dunnett *t* tests or individual *t* tests are used.

To illustrate the procedure, let α=0.025 and assume the unadjusted *p*-values *p*_1_=0.01, *p*_2_=0.02, *p*_3_=0.005, and *p*_4_=0.5. Following Algorithm 3, we have 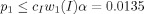 and can reject *H*_1_. The update step then leads to the weights in Figure 2(A). Next, 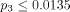 and we can reject *H*_3_. This leaves us with *H*_2_, *H*_4_ and the weights 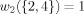, 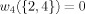. Therefore, *H*_2_ is now tested at full level α. Because *p*_2_≤α, we reject *H*_2_ and the procedure stops.

We now consider the situation that two different populations are used. Assume that the per-protocol population (PP) is used for non-inferiority testing and the intention-to-treat population (ITT) for superiority testing, where PP is a subpopulation of ITT. Let *n*_*i*_ denote the ITT sample size for group *i*, where *i*=0 (1,2) denotes placebo (low dose, high dose). Let further 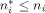 denote the PP sample size for group *i*. Finally, let *T*_*i*_ denote the test statistic for 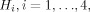 and ρ(*T*_*i*_,*T*_*j*_) the correlation between *T*_*i*_ and *T*_*j*_. With this notation,


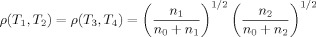


which reduces to 0.5 if *n*_0_=*n*_1_=*n*_2_. Further


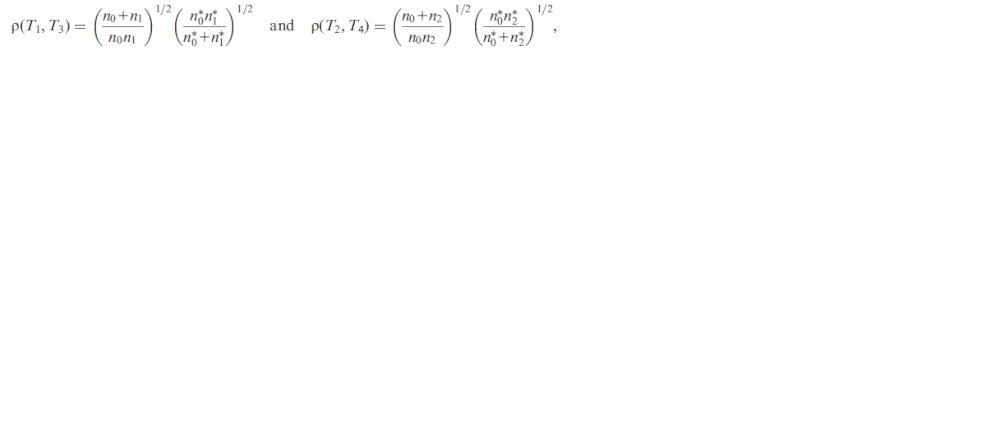


which both reduce to 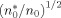 for 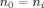 and 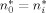, *i*=1,2. Finally, 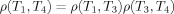 and 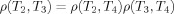, which both reduce to 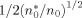 for 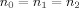 and 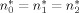. In this simplest case of equal group sample sizes within PP and ITT we thus have, assuming 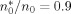 as an example


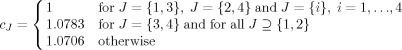


As a consequence, the resulting family of tests is no longer consonant, although the differences in the resulting local significance levels are small. For example, 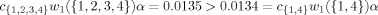, violating condition (4). Similar to Example 2, we can enforce consonance by applying the graphical test procedure from Figure 4 with δ=0.0071.

Finally, we note that this multiple test procedure is immediately applicable to testing for a treatment effect at two different dose levels in an overall population and, if at least one dose is significant, continue testing in a pre-specified subpopulation. This could apply to testing, for example, in the global study population and a regional subpopulation or in the enrolled full population and a targeted genetic subpopulation.

### 3.3 Weighted Simes tests

Generalization of the original Bonferroni-based graphs from Section 3.1 also apply when the correlations between the test statistics are not exactly known, but certain restriction on them are assumed. A typical case in practice is to assume (or show) that the test statistics have a joint multivariate normal distribution with non-negative correlations. In this case, the Simes test is a popular test. Here, we discuss the use of a weighted version of the Simes test for the intersection hypotheses 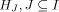.

The unweighted Simes test, as originally proposed by Simes (1986), rejects *H*_*I*_ if there exists a *j*∈*I* such that 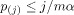, where 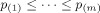 denote the ordered *p*-values for the hypotheses 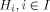. The Type 1 error rate is exactly α if the test statistics are independent and it is bounded by α if positive regression dependence holds. This follows from Benjamini and Yekutieli (2001), who showed false discovery rate control for a related step-up procedure under positive regression dependence on the test statistics. Note that this condition is not always easy to verify or even justify in practice.

The weighted Simes test introduced by Benjamini and Hochberg (1997) rejects *H*_*I*_ if for some *j*∈*I* 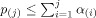, where 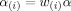 and 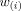 denotes the weight associated with 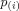. An equivalent condition is to reject *H*_*I*_ if for some *j*∈*I*


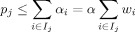
(5)

where 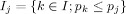. This weighted Simes test reduces to the original (unweighted) Simes test if 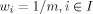. Kling (2005) showed that the weighted test is conservative if the univariate test statistics are positive regression dependent for any number of hypotheses. This, for example, is the case if the test statistics follow a multivariate normal distribution with non-negative correlations and the tests are one-sided (Benjamini and Heller, 2007).

For given weights *w*_*j*_(*J*), *J*⊆*I*, and assuming positive regression dependence among the univariate test statistics for all *m* hypotheses *H*_*i*_, *i*∈*I*, the weighted Simes test can be applied to all intersection hypotheses *H*_*J*_, *J*⊆*I*. By means of the closure principle the resulting multiple test procedure rejects *H*_*i*_, *i*∈*I*, at level α if for each *J*⊆*I* with *i*∈*J*, there exists an index *j*∈*J* such that


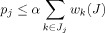
(6)

where 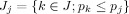. This follows from the application of condition (5) to all subsets *J*⊆*I*, and the fact that any subset of *m* positive regression dependent test statistics is also positive regression dependent. Related gatekeeping procedures based on the Simes tests were described, among others, by Dmitrienko et al. (2003) and Chen et al. (2005).

If all weights are equal, the above procedure reduces to the procedure by Hommel (1988), which is known not to be consonant. In case of unequal weights, a corresponding sequentially rejective test procedure is not available and one may have to go through the entire closed test procedure using weighted Simes tests for each intersection hypotheses. Nevertheless, for a given weighting strategy, this procedure is uniformly more powerful than an associated Bonferroni-based procedure from Section 3.1. This follows from the fact that any hypothesis rejected by the closed weighted Bonferroni test procedure can also be rejected by the corresponding closed weighted Simes test procedure; see, for example, the Appendix in Maurer et al. (2011).

Although full consonance is generally not available for Simes-based closed test procedures, we can still derive a partially sequentially rejective test procedure which leads to the same test decision as the closed test procedure defined in (6). In the following, we assume that the weights are exhaustive, i.e. 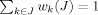 for all subsets *J*∈*I*.

**Algorithm 4 (Weighted Simes Test)**

- If *p*_*i*_>α for all *i*∈I, stop and retain all *m* hypotheses.
- If *p*_*i*_≤α for all *i*∈*I*, stop and reject all hypotheses.
- Perform the Bonferroni-based graphical test procedure from Section 3.1. Let *I*_*r*_ denote the index set of rejected hypotheses and 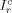 its complement in 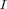. If 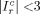, stop and retain the remaining hypotheses.
- If 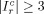 consider the weights 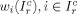, and the transition matrix **G** defined on 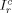 as the new initial graph for the remaining hypotheses. Compute the weights *w*_*k*_(*J*) for all 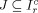 with Algorithm 1.
- Reject 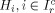, if for each 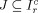 with *i*∈*J*, there exists an index *j*∈*J* such that

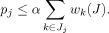
(7)

With step (ii), all hypotheses *H*_*i*_, *i*∈*I* can be rejected if *p*_*j*_≤α for all *j*∈*I*. This follows from the fact that for each *J* there is always a largest *p*_*j*_, *j*∈*J*, such that *J*_*j*_=*J* and therefore 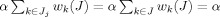. Hence condition (6) holds for all *J*⊆*I* and therefore for all *H*_*i*_, *i*∈*I*. Note that if the weights are not exhaustive, step (ii) may no longer be valid and should be skipped.

The stopping condition in step (iii), 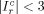, is explained as follows. Assume first that 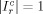, i.e. one hypothesis is left, say *H*_*i*_. If *p*_*i*_<α, one would have rejected already all hypotheses in step (ii) and stopped the procedure because for all other hypotheses than *H*_*i*_ necessarily *p*_*j*_≤α. Therefore, *p*_*i*_>α and one cannot reject *H*_*i*_. Similarly, if 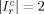, the respective *p*-values cannot be both smaller than *α*. Also if only one of them, say *p*_*i*_, is smaller and the other is larger than α, then 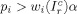, since otherwise the Bonferroni test in step (iii) would have rejected *H*_*i*_. In that case the Simes test cannot reject *H*_*i*_ either and hence both remaining hypotheses must be retained.

Algorithm 4 is essentially looking first for outcomes that are easy to verify (steps (i) and (ii)) or where sequential rejection of the hypotheses is possible (step (iii)). Only then one needs to compute for all remaining hypotheses and their subsets the weights and apply the closed weighted Simes procedure as given in (6). It can happen though that no hypotheses can be rejected in the first three steps and that one has to perform step (iv) with the full set of all *m* hypotheses. Note that one could, of course, start immediately with step (iv) on the full hypotheses set. The resulting decisions are identical to those obtained with Algorithm 4, because for any given weighting strategy, any hypothesis rejected by the closed weighted Bonferroni test procedure is also rejected by the associated closed weighted Simes test procedure.

Similar to the case that knowledge about the joint distribution of the *p*-values is partially missing (as discussed in Section 3.2), we consider now the case that positive regression dependence cannot be assumed between all *m* test statistics. Let 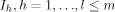, be a a partition (i.e., 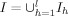 and 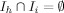 for 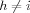) such that for each family of hypotheses 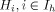, positive regression dependence between the respective test statistics holds. Then we can reject 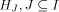, if for some *j* and *h* with 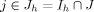



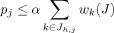
(8)

where 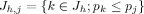. This procedure controls the Type I error rate at level α for any intersection hypothesis *H*_*J*_. This is seen as follows. The weighted Simes test is applied separately to each of the partition sets *J*_*h*_ of *J*. With the definitions for *J*_*h*_ and 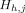 above, for a fixed 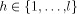, the probability of the event that there exists a *j*∈*J*_*h*_ such that 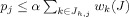, is less than or equal to 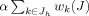 by the weighted Simes test. Hence the probability that this happens in any of the partitions *J*_*h*_ is less than 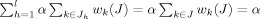 by means of the Bonferroni inequality. For a given partition 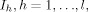 with “local” regression dependence within the disjunct subsets of associated test statistics, condition (7) in the algorithm hence can be replaced by (8).

We conclude this section with an example. For the weighting strategy from Example 1, the resulting closed weighted Simes test will reject more hypotheses than the related closed weighted Bonferroni test only if all four *p*-values are less than or equal to α (Maurer et al., 2011). The latter is not the case for the numerical example in Section 3.1, because, for example, *p*_3_=0.1>0.025=α and hence no further hypothesis can be rejected. However, if we had instead, for example, *p*_3_=0.015 and *p*_4_=0.022, the closed weighted Simes test would reject all four hypotheses, two more than with the closed weighted Bonferroni test. Generally speaking, the weighted Simes test has power advantages over alternative weighted test procedures if the effect sizes are of similar magnitude.

## 4 gMCP package in R

The gMCP package (Rohmeyer and Klinglmueller, 2011) in R (R Development Core Team, 2011) currently implements the Bonferroni-based graphical approach from Section 3.1 and the closed weighted parametric tests from Section 3.2. R is a language and environment for statistical computing and graphics (Ihaka and Gentleman, 1996). It provides a wide variety of statistical and graphical techniques, and is highly extensible. The latest version of gMCP is available at the Comprehensive R Archive Network (CRAN) and can be accessed from http://cran.r-project.org/package=gMCP/. In the following, we give only a brief illustration of the gMCP package. We refer to the installation instructions at http://cran.r-project.org/web/packages/gMCP/INSTALL and the accompanying vignette for a description of the full functionality (Rohmeyer and Klinglmueller, 2011).

### 4.1 Weighted Bonferroni tests with gMCP

We consider the cardiovascular clinical trial example from Dmitrienko and Tamhane (2009) to illustrate the implementation of the Bonferroni-based graphical approach from Section 3.1 in the gMCP package. The trial compared a new compound with placebo for two primary and two secondary endpoints. Consequently, we have two families of hypotheses 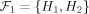 and 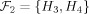.

Dmitrienko and Tamhane (2009) used this example to illustrate the truncated Holm procedure described in Dmitrienko et al. (2008) and Strassburger and Bretz (2008). Given multiple families of hypotheses in a pre-specified hierarchical order, the key idea of truncated tests is to avoid propagating the complete significance level within a family until all its hypotheses are rejected in order to proceed testing the next family in the hierarchy. Instead, once at least one hypothesis is rejected in a given family, a fraction of the significance level is reserved to test subsequent families of hypotheses. In principle, truncation can be applied to any of the test procedures discussed in Section 3.

In the cardiovascular study example, the hypotheses in 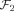 are only tested, if at least one of the hypotheses in 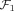 are rejected. We assume that 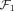 is tested using the truncated Holm procedure with truncation parameter γ∈[0,1]. Let *p*_(1)_<*p*_(2)_ denote the ordered *p*-values with associated hypotheses *H*_(1)_ and *H*_(2)_. Consequently, *H*_(1)_ is tested at level α/2. If *H*_(1)_ is rejected, *H*_(2)_ is tested at level α/2+γ(α/2). The family 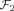 is then tested with the regular Holm procedure either at level (1–γ)α/2 or at level α, depending on whether only one or both hypotheses in 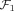 are rejected, respectively.

The gMCP package offers a GUI to conveniently create and perform Bonferroni-based graphical test procedures, such as the one for the test procedure above. To this end, we invoke in R the gMCP package and subsequently call the GUI with

> library(gMCP)

> graphGUI()

**Figure 5:**
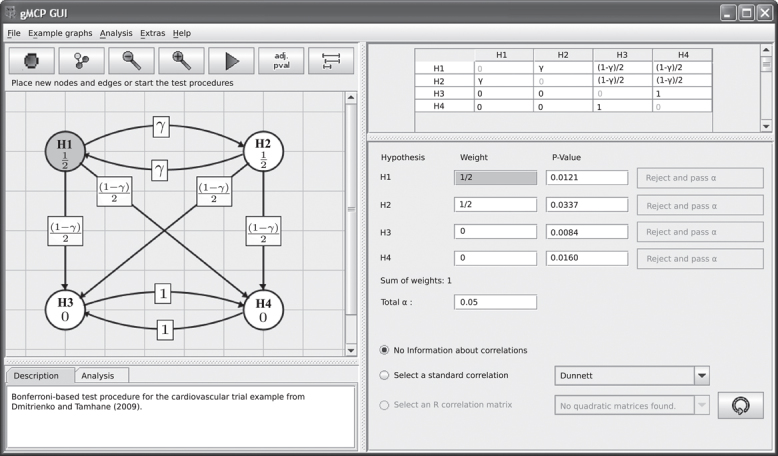
Screenshot of the GUI from the gMCP package. Left: Display of the graphical Bonferroni-based test procedure for the cardiovascular trial. Right: Transition matrix, initial weights and unadjusted *p*-values.

Different buttons are available in the icon panel of the GUI to create a new graph. The main functionality includes the possibility of adding new nodes as well as new edges connecting any two selected nodes. In many cases, the edges will have to be dragged manually in order to improve the readability of the graphs. The associated labels, weights, and significant levels can be edited directly in the graph. Alternatively, the numerical information can be entered into the transition matrix and other fields on the right-hand side of the GUI. Figure 5 displays the complete test procedure for the cardiovascular study example using the gMCP package: The truncated Holm procedure for 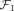 with truncation parameter γ and the regular Holm procedure for 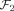. Note that we can immediately improve that test procedure by connecting the secondary hypotheses *H*_3_ and *H*_4_ with the primary hypotheses *H*_1_ and *H*_2_ through the ε-edges introduced in Bretz et al. (2009). We refer to the vignette of the gMCP package for a description of how to construct ε-edges with the GUI (Rohmeyer and Klinglmueller, 2011).

The GUI offers the possibility to perform sequentially Bonferroni-based test procedures defined through a graph like the one displayed in Figure 5 and in addition to calculate adjusted *p*-values as well as simultaneous confidence intervals. To illustrate this functionality, we consider Scenario 1 from Dmitrienko and Tamhane (2009) and assume the unadjusted *p*-values *p*_1_=0.0121, *p*_2_=0.0337, *p*_3_=0.0084, and *p*_4_=0.0160, which are entered directly into the GUI. By clicking on the corresponding button in the icon panel and and specifying γ=0.5, one obtains in this example the adjusted *p*-values 0.024, 0.045, 0.045, and 0.045 for the four hypotheses *H*_1_, *H*_2_, *H*_3_, and *H*_4_, respectively. These adjusted *p*-values are identical to those reported in Dmitrienko and Tamhane (2009). Accordingly, one can reject all four hypotheses at level α=0.05. Simultaneous confidence intervals can be obtained as well from the GUI after entering additional information on effect estimates and standard errors. Finally, the user may perform the sequential test procedure by clicking on the green triangle in the icon bar. By doing so, the “Reject” buttons in the lower right become activated and one can step through the graph as long as significances occur.

### 4.2 Weighted parametric tests with gMCP

The gMCP package provides also a convenient interface to perform graphical test procedures without the GUI using the R command line. We illustrate this with the closed weighted parametric tests from Section 3.2 and revisit Example 2. We first define the related transition matrix **G** and the weights *w*_*i*_(*I*), *i*∈*I*, through

> G <- matrix(0, nr=4, nc = 4)

> G[1,3] <- G[2,4] <- G[3,2] <- G[4,1] <- 1

>w <- c(1/2, 1/2, 0, 0)

The function matrix2graph then converts the matrix G and the vector w into an object of type graphMCP

> graph <- matrix2graph(G, w)

> graph

A graphMCP graph

Overall alpha: 1

H1 (not rejected, alpha=0.5)

H2 (not rejected, alpha=0.5)

H3 (not rejected, alpha=0)

H4 (not rejected, alpha=0)

Edges:

H1 -(1)-> H3

H2 -(1)-> H4

H3 -(1)->H2

H4 -(1)-> H1

The gMCP function takes objects of the type graphMCP as its input together with a vector of *p*-values and performs the specified multiple test procedure. In particular, one can specify a correlation matrix with the effect that a closed weighted parametric multiple test procedure is performed under the standard analysis-of-variance assumptions with known common variance.

In Example 2 we assumed normally distributed test statistics with a block-diagonal correlation matrix of the form


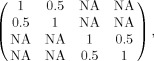


where NA reflects the fact that the correlation between the primary and secondary endpoints is unknown. Accordingly, we let

> cr <- matrix(NA, nr = 4, nc = 4)

> diag(cr) <- 1

> cr[1,2] <- cr[2,1] <- cr[3,4] <- cr[4,3] <- 1/2

and define the unadjusted *p*-values

> p <- c(0.0131, 0.1, 0.012, 0.01)

Finally, we perform the closed weighted parametric test at a specified significance level α=0.025, say, by calling

> res <- gMCP(graph, p, corr = cr, alpha = 0.025)

This returns an object of class gMCPResult providing information on which hypotheses are rejected

> res@rejected H1 H2 H3 H4 TRUE FALSE TRUE FALSE

We conclude from the output that both *H*_1_ and *H*_3_ can be rejected. We come to the same conclusions, if we report the adjusted *p*-values and compare them with α=0.025

> res@adjPValues

H1 H2 H3 H4

0.02431856 0.10000000 0.02431856 0.10000000

Alternatively, one can use a sequentially rejective Bonferroni-based test procedure from Section 3.2 by omitting the corr argument

> gMCP(graph, p, alpha = 0.025)@rejected

H1 H2 H3 H4

FALSE FALSE FALSE FALSE

As seen from the output, none of the null hypotheses can be rejected, which coincides with our conclusions from Section 3.2.

## 5 Discussion

This paper shows that the graphical approach introduced by Bretz et al. (2009) can be used to create and visualize tailored strategies for common multiple test problems. By dissociating the underlying weighting strategy from the employed test procedure, it is seen that the graphical approach is not restricted to Bonferroni-based tests. Similarly, the graphs introduced by Burman et al. (2009) define weights for all intersection hypotheses and the procedures discussed in this paper can be applied using these weights. Extended graphical approaches include weighted Simes tests and weighted min-*p* tests in the sense of Westfall and Young (1993). The latter take into account all or some of the joint multivariate distributions of *p*-values. Consonance and the corresponding shortcuts may be lost, but for any concrete multiple test strategy, consonance can be checked prior to a clinical study. As shown in this paper, consonance can be enforced and related sequentially rejective graphs established at least in some simple situations.

Many proposed multiple test procedures in the literature can be expressed with the methods described in this paper. On the other hand, the methods in this paper also allow one to investigate alternative procedures that go beyond the published results. But even if the closure principle is very common in practice, it does not necessarily lead to consonant multiple test procedures. We gave monotonicity conditions for ensuring consonant graphical weighting strategies, but it is not always clear when these conditions are met if weighted parametric or Simes tests are used. In principle, one could enforce consonance following, for example, the approach of Romano et al. (2011), although the computation of the rejection regions could become tedious. We leave this topic for further research.
